# An Unexpected Finding in a Concussed Circus Acrobat

**DOI:** 10.7759/cureus.37960

**Published:** 2023-04-21

**Authors:** Rock P Vomer, Dusty Narducci, Emma York, Ryan Milon, Imoh Udoh

**Affiliations:** 1 Family Medicine, Mayo Clinic Jacksonville Campus, Jacksonville, USA; 2 Department of Family and Community Health, Department of Orthopaedic Surgery, Division of Sports Medicine, Duke University, Durham, USA; 3 Family Medicine, University of South Florida Morsani College of Medicine, Tampa, USA; 4 Family Medicine, Eastern Virginia Medical School, Norfolk, USA; 5 Family and Community Medicine, Mayo Clinic Jacksonville Campus, Jacksonville, USA; 6 Department of Orthopaedic Surgery, Division of Sports Medicine, Duke University, Durham, USA

**Keywords:** neurosyphilis, sexually transmitted infections, syphillis, concussion, persistent post concussive syndrome

## Abstract

Persistent post-concussive syndrome (PPCS) outlines a complex array of neurocognitive and psychological symptoms that persist in patients after a concussion. A 58-year-old female presented reporting recurrent loss of consciousness, and retrograde and anterograde amnesia following multiple concussions. She also endorsed persistent nausea, balance insufficiencies, hearing loss, and cognitive impairment. In addition, this patient had high-risk sexual behavior without prior testing for sexually transmitted infections. Given her clinical history, the differential included PPCS, complex post-traumatic stress disorder, Korsakoff syndrome, hypothyroidism, and sexually transmitted infection (STI)-related neurocognitive disorder. On exam, this patient had a positive Romberg sign, prominent resting tremoring of upper extremities, and pinpoint pupils unresponsive to light, with bilateral nystagmus. Syphilis testing was positive. The patient was treated with intramuscular benzathine penicillin with significant improvement in gait, balance, headaches, vision, and cognition three months after treatment. Although rare, neurocognitive disorders, including late-stage syphilis, should be considered in the differential diagnosis for PPCS.

## Introduction

Concussion, the most common form of traumatic brain injury, can produce a constellation of physical, cognitive, behavioral, and/or emotional symptoms in an affected individual [1]. Although concussion symptoms frequently resolve within one to two weeks, persistent post-concussive syndrome (PPCS) previously termed post-concussion syndrome (PCS) defines symptoms that persist beyond this time [2]. Classically, depression, fibromyalgia, post-traumatic stress disorder (PTSD), vertebral artery disease, headache syndromes, and exposure to certain toxins are the most commonly considered differential diagnoses for PPCS, with sexually transmitted infections (STI) such as late-stage syphilis rarely considered.

## Case presentation

Patient presentation

A 58-year-old cis female presented to a university-based concussion center reporting a complex history of approximately 24 head injuries beginning at age two with the most recent occurring three years prior. She described direct and indirect forms of head trauma from various mechanisms including motor vehicle accidents and sport-related injuries involving participation in soccer and circus acrobatics. To the best of her ability, she recalled an associated loss of consciousness, retrograde and anterograde amnesia following several of these injuries. She portrayed significant distress regarding symptoms of persistent nausea, fatigue, headaches, mood changes, balance insufficiencies, hearing loss, and cognitive impairment. Her ability to provide an accurate and detailed medical history was limited by her memory. Treatment for her reported concerns has been limited to non-prescription medication for headache relief in addition to unsuccessful psychotherapy and psychopharmaceutic management of mood symptoms.  

Reported medical history consisted of PTSD, pancreatitis, gastritis, irritable bowel syndrome, untreated Hashimoto's thyroiditis, lumbar and cervical spine pathology requiring surgical interventions, and daily narcotic medications for pain control. She indicated prior high-risk sexual behavior with no prior testing for sexually transmitted infections. She stated that she was employed as a math professor and traveling circus performer prior to entering retirement 10 years ago. She denied recent use but acknowledged prior intermittent use of cannabinoids, tobacco, and alcohol.

Physical exam

Examination demonstrated a thin body habitus, with no obvious deformity or atrophy. Blood pressure was 147/90 and heart rate was 60 beats per minute. Supine, standing, and sitting blood pressure and heart rate measurements were similar. Ambulatory assessment revealed an unstable gait with increased stance width. Although muscle strength and joint range of motion were grossly normal, Romberg sign was positive. She had prominent resting tremoring of her upper extremities, pinpoint pupils unresponsive to light, with bilateral nystagmus. Neurocognitive computerized testing showed impairment in visual more than verbal memory and slowed reaction and motor speed. Patient Health Questionnaire-9 (PHQ-9) and the General Anxiety Disorder-7 (GAD-7) instruments administered were 27 and 19 respectively.

Differential diagnosis

The differential diagnosis was broad for this patient presentation, and included neurologic, metabolic, endocrine conditions, and substance ingestion. The differential diagnosis list included the following: PPCS, complex PTSD, Korsakoff syndrome, hypothyroidism or hyperthyroidism, and STI-related neurocognitive disorder. 

Imaging and diagnostics

To assess the wide array of symptoms and clinical findings, a broad array of laboratory tests were utilized to determine possible etiologies. A complete blood count (CBC) with differential, complete metabolic profile (CMP), urine drug screen (UDS), thiamine, vitamin B-12, folate, thyroid panel, hepatitis C, human immunodeficiency virus (HIV), thyroid peroxidase (TPO) antibodies, rapid plasma regain (RPR), and venereal disease research laboratory (VDRL) were within normal limits. Treponema pallidum particle agglutination assay (TPPA) was positive on two occasions. Cerebral spinal fluid (CSF) demonstrated lymphocytic pleocytosis, elevated protein, negative VDRL, and nonreactive Treponema pallidum particle agglutination (TP-PA).

**Figure 1:**
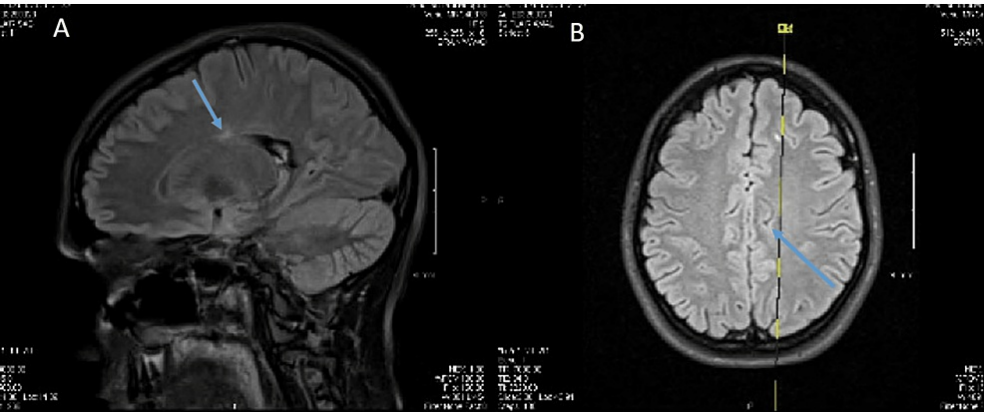
A (Sagittal) and B (Transverse) MRI demonstrating numerous small, non-enhancing periventricular and juxtacortical T2 hyperintensities (arrow).

Audiometry demonstrated bilateral sensorineural hearing loss, an echocardiogram revealed no abnormalities, and magnetic resonance imaging (MRI) of the brain with and without contrast showed chronic white matter changes with volume loss more than expected for the patient's chronological age (Figure 1).

Treatment

Given subjective and objective clinical findings, history of high-risk sexual behavior and unclear health history, the consulting infectious disease physician recommended treatment for latent syphilis. The patient agreed and was provided three doses of intramuscularly administered benzathine penicillin at 2.4 million units weekly.

Outcome

Three months following treatment she reported significant improvement in gait, balance, headaches, vision, and cognition, but continued to report severe mood symptoms. Serum RPR remained negative at six, 12, and 24 months following treatment. She returned to walking for exercise and discontinued daily use of narcotic medications for the alleviation of pain, however, she remains under the care of multiple mental health professionals to manage her psychiatric symptoms.

## Discussion

Healthy persons, appropriately determined to be concussed, often report symptom resolution within seven to 14 days from the time of injury [1,2]. Although no universally accepted criteria for diagnosis exist in the literature, PPCS, formerly termed PCS, outlines a complex array of unrelenting neurocognitive and psychological symptoms following a concussion [3]. According to the Diagnostic and Statistical Manual of Mental Disorders, Fifth Edition (DSM-IV), criteria of PCS require symptoms to be present for longer than three weeks whereas symptoms beyond three months is preferred by most experts in the field [4,5]. Both PPCS and PCS are controversial given vague, subjective symptoms, no accepted terminology, and inconsistent research findings. Although the underlying pathophysiology is undefined, multiple biochemical and structural changes are suspected. Pathological psychogenic factors are assumed to contribute to many of the reported symptoms. In theory, trauma to the cerebral white matter tracks disrupt communication between the brain and the spinal cord, resulting in dysregulation of the autonomic nervous system [6]. When the parasympathetic and sympathetic branches of the autonomic nervous system are not functioning properly, symptoms consistent with depression, and other mood conditions can occur [7]. A change in cerebral blood flood as well as other less understood physiological adaptations can cause somatic changes in blood pressure, heart rate, and metabolism with affected individuals often reporting dizziness, headache, confusion, and difficulties with balance, sleep, and vision [8]. Our current literature includes depression, fibromyalgia, PTSD, vertebral artery disease, headache syndromes, sleep disorders, exposure to certain toxins, and other neurocognitive disorders as differential diagnoses for PPCS. Sexually transmitted infections including late-stage syphilis or acquired immunodeficiency syndrome (AIDS) are rarely considered as a differential diagnosis of PPCS [9].

Approximately 10% of untreated syphilis cases result in brain and nervous system pathology [6,8]. Unlike primary, secondary, and tertiary syphilis, involvement of the central nervous system, termed neurosyphilis (NS), can occur at any time after infection. NS can lead to symptoms including cognitive impairment, ataxia, aphasia, vision changes, neuropathy, hearing loss, inability to control bowel or bladder function, seizures, hyperreflexia, and many others [8]. These signs and symptoms mimic other medical and neuropsychiatric disorders, including personality disorders, psychosis, dementia, and as seen in this case PPCS. NS can develop decades after the original infection making it difficult to diagnose and provide patients with the best opportunity for a positive health outcome [8].

There are two sequelae of late neurosyphilis to consider, general paresis and tabes dorsalis. General paresis is a consequence of syphilitic encephalitis that leads to inflammation and diffuse demyelination of the tissues covering the brain and spinal cord. Rapid cognitive impairment is the common presenting symptom of syphilitic encephalitis. Tabes dorsalis is characterized by demyelination of the optic nerve, dorsal column and dorsal root of the spinal cord which functions to maintain a person’s sense of position. Progressive parenchymatous degeneration of the dorsal roots and posterior columns seen in tabes dorsalis causes paresthesia, neuropathic pain, sensory ataxia, impaired pupillary constriction in response to light but pupils constrict to near objects (Argyll Roberston pupils), and optic atrophy among many other manifestations [5].

Since no standardized testing for NS exists, diagnosis is challenging and relies on patient history, examination, and clinical and laboratory findings. Prior to considering a diagnosis of NS there must be a significant suspicion or known syphilis infection. CSF testing is performed to analyze reactivity to CSF VDRL, protein count and white blood cell count (WBC). The Centers for Disease Control and Prevention (CDC) delineates confirmed and presumptive neurosyphilis diagnoses when CSF is these results for patients with syphilis: a confirmed case is defined as any stage of syphilis with a positive VDRL test, whereas a presumptive case must include any stage of syphilis, a negative VDRL, elevated protein or WBC count in CSF without other causes for these elevations, or clinical signs/symptoms for neurosyphilis without other present causes [5]. This patient had a negative RPR and positive TPPA indicating either a treated past infection or late syphilis. Although CSF testing found VDRL to be negative, a high protein and leukocyte count with symptoms and no confirmed etiology made NS more likely, prompting the infectious disease specialist to recommend treatment for tertiary syphilis.

## Conclusions

This case illustrates one of several challenges in diagnosing PPCS as well as NS. Additional care must be taken to ascertain an accurate diagnosis when a patient presents with an incongruent timeline, unclear history and other concerning clinical findings. Although rare, neurocognitive disorders including late-stage syphilis should be considered in the differential diagnosis for PPCS. Increased clinical awareness and future studies may reveal a higher incidence of sexually transmitted infections in the diagnosis of PPCS.
